# Molecular and biophysical features of hippocampal “lipid rafts aging” are modified by dietary n‐3 long‐chain polyunsaturated fatty acids

**DOI:** 10.1111/acel.13867

**Published:** 2023-05-30

**Authors:** Mario Díaz, Daniel Pereda de Pablo, Catalina Valdés‐Baizabal, Guido Santos, Raquel Marin

**Affiliations:** ^1^ Department of Physics, Faculty of Sciences University of La Laguna Tenerife Spain; ^2^ Instituto Universitario de Neurociencias (IUNE) Tenerife Spain; ^3^ Laboratory of Membrane Physiology and Biophysics, School of Sciences University of La Laguna Tenerife Spain; ^4^ Laboratory of Cellular Neurobiology Department of Basic Medical Sciences, Faculty of Health Sciences University of La Laguna Tenerife Spain; ^5^ Department of Biochemistry, Microbiology, Cellular Biology and Genetics, School of Sciences University of La Laguna Tenerife Spain; ^6^ Associate Research Unit ULL‐CSIC “Membrane Physiology and Biophysics in Neurodegenerative and Cancer Diseases” Tenerife Spain

**Keywords:** acid‐sensing ion channels, Aging, AMPA‐R, arachidonic acid, cholesterol, DHA, dietary LCPUFA, fluidity, ganglioside GM1, lipid rafts, membrane microdomains, mGlu‐R, microviscosity, NMDA‐R, sphingolipids

## Abstract

“Lipid raft aging” in nerve cells represents an early event in the development of aging‐related neurodegenerative diseases, such as Alzheimer's disease. Lipid rafts are key elements in synaptic plasticity, and their modification with aging alters interactions and distribution of signaling molecules, such as glutamate receptors and ion channels involved in memory formation, eventually leading to cognitive decline. In the present study, we have analyzed, in vivo, the effects of dietary supplementation of n‐3 LCPUFA on the lipid structure, membrane microviscosity, domain organization, and partitioning of ionotropic and metabotropic glutamate receptors in hippocampal lipid raffs in female mice. The results revealed several lipid signatures of “lipid rafts aging” in old mice fed control diets, consisting in depletion of n‐3 LCPUFA, membrane unsaturation, along with increased levels of saturates, plasmalogens, and sterol esters, as well as altered lipid relevant indexes. These changes were paralleled by increased microviscosity and changes in the raft/non‐raft (R/NR) distribution of AMPA‐R and mGluR5. Administration of the n‐3 LCPUFA diet caused the partial reversion of fatty acid alterations found in aged mice and returned membrane microviscosity to values found in young animals. Paralleling these findings, lipid rafts accumulated mGluR5, NMDA‐R, and ASIC2, and increased their R/NR proportions, which collectively indicate changes in synaptic plasticity. Unexpectedly, this diet also modified the lipidome and dimension of lipid rafts, as well as the domain redistribution of glutamate receptors and acid‐sensing ion channels involved in hippocampal synaptic plasticity, likely modulating functionality of lipid rafts in memory formation and reluctance to age‐associated cognitive decline.

## Graphical abstract

“Lipid rafts aging” has been described as a phenomenon associated with nerve cell membranes during normal brain aging, which is exacerbated in pathological processes leading to neurodegeneration. Dietary n‐3 long‐chain polyunsaturated fatty acids (n‐3 LCPUFA) partly revert the molecular and biophysical alterations of hippocampal lipid rafts associated with aging. Besides membrane lipidome and microviscosity, the diet also modified lipid rafts dimensions and the membrane distribution of glutamate receptors and acid‐sensing ion channels involved n hippocampal synaptic plasticity.

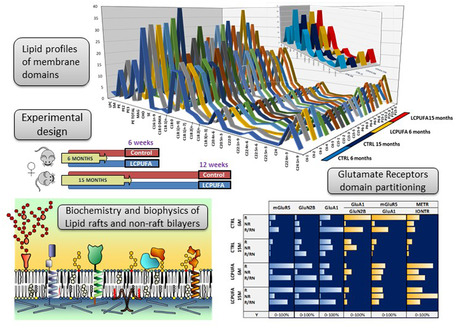

**Abbreviations**
AA: Arachidonic acid, 20:4n‐6
AMPA‐R: α‐amino‐3‐hydroxy‐5‐methyl‐4‐isoxazolepropionic acid receptor subtype
AN/ZW: Anionic to Zwitterionic phospholipids ratio
ASIC: Acid‐sensing ion channel
CB: Cerebrosides
CHO: Cholesterol
DHA: Docosahexaenoic acid, 22:6n‐3
DMA: Dimethyl acetals
DPA: Docosapentaenoic acid, 22:5n‐6
DPH: Diphenylhexatriene
EPA: Eicosapentaenoic acid, 20:5n‐3
FLOT‐1: Flotillin‐1
GM1: Ganglioside GM1
GSL: Glycosphingolipid
LCPUFA: Long‐chain polyunsaturated fatty acids
LPC: Lysoglycerophosphatidylcholine
MAG: Monoacylglycerol
METR/IONTR: Metabotropic to ionotropic receptors ratio
mGluR5: Metabotropic glutamate receptor subtype 5
MUFA: Monounsaturated fatty acids
NMDA‐R: N‐methyl‐D‐aspartate receptor
PC: Glycerophosphatidylcholine
PE: Glycerophosphatidylethanolamine
PG: Glycerophosphatidylglycerol
PI*: Peroxydability index
PI: Glycerophosphatidylinositol
PISAT: Peroxydability index to saturated fatty acids ratio
PlsEtn: Glycerophosphatidylethanolamine plasmalogen
R/NR: Raft‐to‐non‐raft proportion
ROS: Reactive oxygen species
SE: Steryl esters
SF: Sulphatides
SFA: Saturated fatty acids
SM: Sphyngomyelin
TMA‐DPH: Trimethylammonium‐diphenylhexatriene
TNL: Total neutral lipids
TPE: Total diacylglycerophosphatidylethanolamine
TPL: Total polar lipids
UI: Unsaturation index
UISAT: Unsaturation index to saturated fatty acid ratio

## 1 INTRODUCTION

The brain tissue exhibits the most complex lipidome in the whole body (Mota‐Martorell et al., 2022; Naudí et al., 2015; Yoon et al., 2022). Unique in the brain tissue, it is the fact that nearly all lipids are contained in plasma and intracellular membranes, which, in turn, are organized in highly dynamic microdomains. A particular type of membrane microdomains named lipid rafts has received considerable attention. Lipid rafts are liquid‐ordered domains enriched in membrane lipids such as cholesterol, sphingolipids, saturated acyl chains, and gangliosides (GM1), which create a physicochemical environment for molecular interactions favorable for the compartmentalization of protein clusters governing signaling pathways (Egawa et al., 2016; Lorent & Levental, 2015; Suzuki et al., 2011). The molecular clusters are altered in response to pathophysiological conditions, such as oxidative stress and neurodegeneration (Allen et al., 2007; Díaz & Marin, 2021; Marin & Diaz, 2018).

Recent studies have shown that NMDA receptors (NMDARs) activation in the hippocampus involves translocation to synaptic lipid rafts during plasticity processes necessary for spatial memory formation (Bannerman et al., 2014). Spatial training induces a rapid and selective translocation of NR1 and NR2A NMDA‐R subunits and scaffolding PSD‐95 to synaptic lipid rafts and decreases the soluble fraction of postsynaptic membrane (non‐raft PSD) (Delint‐Ramirez et al., 2010). Furthermore, lateral reallocation of AMPA receptors affecting synaptic strength has been demonstrated between synaptic nanodomains under experimental short‐ and long‐term potentiation (STP and LTP) conditions (Groc & Choquet, 2020).

Age‐related changes in both total lipid abundance and region‐dependent composition have long been observed (Colin et al., 2016; Ledesma et al., 2012; Ulmann et al., 2001; Wood, 2012). The brain shows a high (~20%) content of n‐3 and n‐6 long‐chain polyunsaturated fatty acids (LCPUFAs), in particular, docosahexaenoic acid (DHA, C22:6n‐3) and arachidonic acid (AA, C20:4n‐6) (Naudí et al., 2015). Dietary omega‐3 fatty acids are actively retained in central nervous system membranes, mainly in synapses and dendrites. LCPUFA content has generally been observed to drop (unevenly) during aging in different brain areas. This may be caused by altered fatty acid metabolism (including membrane phospholipid biogenesis), lower rate of transport of LCPUFAs through the blood–brain barrier, and peroxidation of LCPUFA pools (Catalá & Díaz, 2016; Fabelo et al., 2014; Naudí et al., 2015; Yoon et al., 2022). A wealth of studies on aging‐associated changes in brain lipids indicate that besides LCPUFA depletion, other important lipid components of nerve cell membranes are also altered in an age‐ and region‐specific manner (Colin et al., 2016; Ledesma et al., 2012; Naudí et al., 2015). Neurolipids reported to be affected by aging include ethanolamine glycerophospholipids and plasmalogens, GM1 and GM3 gangliosides, sulfatides, sphingolipids, ceramides, cholesterol and cholesterol esters (Brites et al., 2004; Chiricozzi et al., 2020; Favrelière et al., 2000; Naudí et al., 2015; Ulmann et al., 2001).

Although much less explored, brain lipid rafts undergo complex lipid modifications associated with aging, which likely impact synaptic transmission. We have previously described a complex set of changes in the contents of different fatty acids, lipid classes, and biophysical properties in brain cortex lipid rafts from male mice associated with aging (Diaz et al., 2012). This phenomenon, termed “lipid raft aging”, was subsequently demonstrated in lipid rafts from the human frontal cortex (Díaz et al., 2018; Fabelo et al., 2014). Lipid raft aging is characterized by changes in the lipid structure of the raft bilayer are caused by a marked reduction in membrane unsaturation and peroxydability and cholesterol‐to‐phospholipids ratio, increased sphingolipids (sphingomyelin and sulfatides), saturated fatty acids, and sterol esters (Fabelo et al., 2012), which collectively render lipid rafts more viscous and less fluid (Diaz et al., 2012; Díaz et al., 2016).

There exists considerable interest in the possible role of dietary fatty acids in age‐related cognitive decline and cognitive impairment related to Alzheimer's disease, raising the possibility that dietary LCPUFA may be incorporated as nutraceuticals for the prevention of age‐associated neuropathologies. Indeed, several observational and epidemiological studies have suggested that saturated fatty acids have negative effects on cognitive function and that dietary intakes of PUFAs are associated with reduced risk for age‐associated cognitive decline and protection against the risk of AD (Solfrizzi et al., 2018; Zhang et al., 2020). Moreover, experimental evidence has shown that nerve cell membrane LCPUFA may be modified by dietary interventions (Díaz et al., 2016; Yaqoob & Shaikh, 2010). However, one common observation from nutritional studies is that both, diets deprived of, or enriched in, LCPUFA or their precursors, have a rather discrete (yet significant) effect on the overall brain lipid composition, despite severe nutritional interventions. This clearly limits the efficiency of dietary manipulations to correct age‐associated depletion in essential fatty acids and related lipid classes but also demonstrates the existence of highly efficient lipostatic mechanisms preserving nerve cell membrane functionality. Nevertheless, the extent to which LCPUFA content affects the neuronal membrane, remains largely unexplored, especially in the context of synaptic plasticity and memory formation. This is particularly relevant in females, where the presence of ovarian hormones are modulatory factor for hepatic and brain lipid metabolism, and because nerve cells lipidome is subjected to a complex interplay between estrogens and endogenous biosynthesis and/or dietary LCPUFA availability (Díaz et al., 2016).

In the present study, we sought to investigate changes in lipid profiles, physicochemical properties, and distribution of synaptic receptors in hippocampal lipid rafts during aging. We have used cycling female mice. Estrogens are known to modulate the effects of dietary LCPUFAs on the cerebral cortex (Díaz et al., 2016) and estrogen decline is associated with a higher incidence of neurological disorders (Marin & Diaz, 2018). We have undertaken a heuristic approach based on global lipid profiles. This allowed us to assess the relevance of lipid variations associated with normal aging in hippocampal lipid rafts. Also, we have investigated the compensatory effects of n‐3 LCPUFA supplementation to counteract the effects of aging on lipid raft structure and functionality. The outcomes have disclosed age‐independent modifications on the lipid signature, interdomain remodeling, biophysical properties, and glutamate receptor distribution in female hippocampal lipid rafts in response to n‐3 LCPUFA supplementation.

## 2 RESULTS AND DISCUSSION

### 2.1 Aging modifies the lipid composition of Hippocampal lipid rafts

The composition of lipid classes and fatty acids extracted from hippocampal lipid raft fractions in the four experimental conditions are shown in Tables 1 and 2. Lipid profile in 6 months control animals (CTRL6M) fed a standard diet display a close agreement with lipid classes and fatty acid data previously reported for hippocampal lipid rafts under control conditions (Canerina‐Amaro et al., 2019; Diaz et al., 2012; Fabelo et al., 2012).

**TABLE 1:** Representative fatty acid composition, indexes and ratios obtained in hippocampal lipid rafts in the different groups. Results from two‐way ANOVA for main factors and their interactions on each variable is summarized in the right panel.

Fatty acids	6M Control		15M Control		6M LCPUFA		15M LCPUFA		Factors and interactions					
	Mean ± SD	SIG	Mean ± SD	SIG	Mean ± SD	SIG	Mean ± SD	SIG	Diet		Age		Diet*Age	
C14:0	0.67 ± 0 23		0.61 ± 0.11		0.45 ± 0.16		0.46 ± 0.15			NS		NS		NS
C14:1(n‐5)	0.08 ± 0.17		0.20 ± 0.45		0.33 ± 0.74		0.23 ± 0.51			NS		NS		NS
C15:0	0.89 ± 0.77		0.73 ± 0.27		0.58 ± 0.54		0.40 ± 0.23			NS		NS		NS
C16:0 DMA	1.32 ± 0.40		1.00 ± 0.35		1.36 ± 0.40		1.30 ± 0.14			NS		NS		NS
C16:0	26.91 ± 2.18		29.78 ± 3.67		27.65±4.32		29.99 ± 2.23		3.16	0.09		NS		NS
C16:1n‐9	0.49 ± 0.13		0.44 ± 0.07		0.27 ± 0.27		0.47 ± 0.07			NS		NS		NS
C16:1(n‐7)	0.91±0.33		0.61 ± 0.08		0.59±0.12		0.78 ± 0.24			NS		NS		NS
C18:0 DMA	1.95 ± 0.41		2.05±0.13		2.30 ± 0.34		2.29 ± 0.30			NS		NS		NS
C18:1(n‐9) DMA	0.47 ± 0.11		0.51 ± 0.09		0.38 ± 0.22		0.49 ± 0.07			NS		NS		NS
C18:1(n‐7) DMA	0.64 ± 0.14		0.67 ± 0.13		0.61 ± 0.04		0.70 ± 0.13			NS		NS		NS
C18:0	18.45 ± 1.20		22.36 ± 2.84		19.97 ± 3.22		19.31 ± 1.62			NS		NS	4.23	0.06
C18:1(n‐9)	14.87 ± 1.15		12.45 ± 1.98		13.74 ± 0.79		13.24 ± 1.68			NS		NS		NS
C18:1(n‐7)	3.44 ± 0.23		3.38 ± 0.43		3.26 ± 0.69		3.30 ± 0.53			NS		NS		NS
C18:2(n‐6) cis	1.22 ± 0.79	*a*	0.81 ± 0.75	*a*	0.69 ± 0.87	*a*	0.13 ± 0.30	*b*	3.21	0.09		NS		NS
C18:2(n‐6)trans	0.16 ± 0.35	*b*	0.42 ± 0.60	*a*	0.40 ± 0.54	*a*	0.69 ± 0.43	*a*		NS		NS		NS
C18:2(n‐6)	1.38 ± 0.57		1.23 ± 0.68		1.09 ± 0.71		0.82 ± 0.36			NS		NS		NS
C18:3(n‐4)	0.21 ± 0.31	**ab**	0.09 ± 0.21	**ab**	0.50 ± 0.34	**a**	0.00 ± 0.00	**b**		NS	7.63	0.01		NS
C18:3(n‐3)	0.23 ± 0.22	*a*	0.14 ± 0.19	*b*	0.11 ± 0.24	*b*	0.00 ± 0.00	*b*	3.23	0.09		NS		NS
C18:4(n‐3)	0.00 ± 0.00	**b**	0.00 ± 0.00	**b**	0.14 ± 0.32	**a**	0.14 ± 0.21	**a**	3.24	0.09		NS		NS
C20:0	0.34 ± 0.02		0.38 ± 0.03		0.25 ± 0.14		0.22 ± 0.12			NS		NS		NS
C20:1n‐9	1.03 ± 0.38		0.76 ± 0.29		0.98 ± 0.45		1.15 ± 0.54			NS		NS		NS
C20:1n‐7	0.16 ± 0.15		0.28 ± 0.05		0.34 ± 0.15		0.19 ± 0.11			NS		NS		NS
C20:3n‐6	0.00 ± 0.00	**b**	0.22 ± 0.34	**a**	0.21 ± 0.19	**a**	0.27 ± 0.15	**a**	4.02	0.06		NS		NS
C20:4n‐6	5.85 ± 0.36	**a**	5.00 ± 0.39	**ab**	4.97 ± 0.89	**ab**	4.76 ± 0.54	**b**	4.61	0.05	4.14	0.06		NS
C20:5n‐3	1.14 ± 0.98		0.76 ± 0.60		0.65 ± 0.94		0.79 ± 0.51			NS		NS		NS
C22:1n‐9	0.27 ± 0.39		0.29 ± 0.30		0.28 ± 0.18		0.35 ± 0.26			NS		NS		NS
C22:4n‐6	1.55 ± 0.06	*a*	1.41 ± 0.20	*ab*	1.20 ± 0.09	*bc*	1.07 ± 0.10	*c*		NS		NS		NS
C22:5n‐6	0.24 ± 0.25	**a**	0.06 ± 0.14	**a**	0.00 ± 0.00	**b**	0.00 ± 0.00	**b**	6.35	0.02		NS		NS
C22:5n‐3	0.17 ± 0.23		0.10 ± 0.14		0.29 ± 0.16		0.37 ± 0.07		6.96	0.02		NS		NS
C24:0	0.34 ± 0.19		0.20 ± 0.31		0.47 ± 0.30		0.22 ± 0.22			NS	3.82	0.07		NS
C22:6n‐3	8.64 ± 1.43	**a**	6.33 ± 0.55	**b**	9.22 ± 1.72	**a**	10.10 ± 1.07	**a**	14.69	0.00		NS	7.90	0.01
C24:1n‐9	1.15 ± 0.56		0.77 ± 0.34		0.91 ± 0.51		1.50 ± 0.93			NS		NS	3.33	0.09
*Totals. indexes and ratios*														
n‐3	10.17 ± 2.51	**a**	7.33 ± 0.69	**b**	10.45 ± 1.52	**a**	11.40 ± 1.05	**a**	9.25	0.01		NS	7.07	0.02
n‐6	8.71 ± 1.47		8.08 ± 0.97		7.47 ± 0.90		6.91 ± 0.61		6.81	0.02		NS		NS
n‐9	17.32 ± 0.73	**a**	14.28 ± 2.42	**b**	15.90 ± 1.60	**ab**	16.24 ± 1.40	**ab**		NS	3.35	0.09	5.22	0.04
n‐6 /n‐3	0.92 ± 0.32	**ab**	1.10 ± 0.10	**a**	0.72 ± 0.05	**b**	0.61 ± 0.06	**b**	19.96	0.00		NS		NS
Saturates	47.87 ± 2.91		54.39 ± 6.26		49.65 ± 6.81		50.99 ± 3.54			NS		NS		NS
Monoenes	22.64 ± 1.21		19.71 ± 3.12		21.02 ± 2.91		21.55 ± 2.04			NS		NS		NS
PUFA	18.89 ± 2.02	**a**	15.41 ± 1.50	**b**	17.92 ± 2.38	**ab**	18.31 ± 1.36	**ab**		NS	3.41	0.08	5.40	0.03
LCPUFA	17.28 ± 2.24	**a**	13.88 ± 1.44	**b**	16.58 ± 2.59	**ab**	17.35 ± 1.69	**ab**		NS		NS	5.21	0.04
PUFA/SFA	0.40 ± 0.06	**a**	0.29 ± 0.05	**b**	0.37 ± 0.08	**ab**	0.36 ± 0.02	**ab**		NS	5.69	0.03	4.28	0.06
UI	114.76 ± 12.71	**a**	92.09 ± 6.60	**b**	111.11 ± 11.09	**a**	114.41 ± 7.89	**a**	4.47	0.05	4.80	0.04	8.64	0.01
PI	109.64 ± 16.06	**a**	84.60 ± 7.57	**b**	108.14 ± 15.32	**a**	113.63 ± 10.13	**a**	5.80	0.03		NS	7.14	0.02
UISAT	2.41 ± 0.34	**a**	1.72 ± 0.29	**b**	2.28 ± 0.42	**a**	2.25 ± 0.16	**a**	6.39	0.02		NS	5.43	0.03
DMA	4.38 ± 0.32		4.23 ± 0.62		4.65 ± 0.85		4.78 ± 0.50			NS		NS		NS

*Note*: Data are expressed as mean ± SD for 5–6 animals per group. Different letters in each row indicate statistical differences at *p* < 0.05 (bold) or 0.05 < *p* < 0.1 (italic). n‐3, n‐6 and n‐9: first double‐bond series.

Abbreviations: DMA, Dimethylacetals; LCPUFA, Long‐chain PUFA; PI, peroxydability index; PUFA, polyunsaturated fatty acids; SFA, saturated fatty acids; UI, Unsaturation index; UISAT, UI to saturates ratio.

**TABLE 2:** Lipid classes composition, indexes and ratios obtained in hippocampal lipid rafts in the different groups. Results from two‐way ANOVA for main factors and their interactions on each lipid variable is summarized in the right panel.

Lipid classes	6M Control		15M Control		6M Lcpufa		15M Lcpufa		Factors and interactions					
	Mean ± SD	SIG	Mean ± SD	SIG	Mean ± SD	SIG	Mean ± SD	SIG	Diet		Age		Diet*Age	
LPC	0.86 ± 1.36	**a**	1.34 ± 0.34	**a**	0.72 ± 1.61	**b**	3.20 ± 3.05	**a**	4.26	0.05	6.51	0.02	6.56	0.02
SM	5.51 ± 2.65	**ab**	2.34 ± 0.85	**b**	6.35 ± 1.97	**a**	3.27 ± 1.62	**b**		NS	16.26	0.00		NS
PC	10.27 ± 1.01		9.23 ± 4.73		10.83 ± 2.30		11.31 ± 3.01			NS		NS		NS
PS	6.03 ± 0.50		9.43 ± 3.30		7.20 ± 1.22		6.63 ± 1.91			NS		NS		NS
PI	2.26 ± 0.21		2.67 ± 0.90		3.14 ± 1.69		2.63 ± 0.92			NS		NS		NS
PG	2.05 ± 1.37		2.17 ± 1.23		1.97 ± 1.43		1.77 ± 1.12			NS		NS		NS
PE	16.01 ± 2.67		16.74 ± 2.01		17.07 ± 4.29		14.33 ± 1.65			NS		NS		NS
PlsEtn(a)	1.31 ± 1.28	*b*	3.02 ± 1.46	*a*	1.53 ± 0.95	*b*	2.63 ± 0.31	*ab*		NS	4.10	0.06		NS
PlsEtn(b)	1.23 ± 1.44	**ab**	0.16 ± 0.28	**b**	2.03 ± 1.36	**a**	2.42 ± 1.50	**a**	9.08	0.01		NS		NS
Total PE (TPE)	18.55 ± 1.02		19.92 ± 2.69		20.63 ± 2.75		19.38 ± 2.87			NS	4.65	0.04		NS
SF	7.85 ± 3.05		7.86 ± 1.65		7.22 ± 2.23		6.93 ± 1.49			NS		NS		NS
CB	2.86 ± 1.88		3.05 ± 0.76		2.77 ± 1.41		2.70 ± 0.65			NS		NS		NS
MAG	5.61 ± 0.91		5.24 ± 2.21		5.15 ± 1.48		4.22 ± 1.63			NS		NS		NS
CHO	33.03 ± 1.28		28.28 ± 7.39		26.60 ± 9.62		31.73 ± 4.36			NS		NS		NS
SE	5.13 ± 4.56	*b*	7.28 ± 3.71	*b*	6.24 ± 4.04	*b*	6.24 ± 2.31	*ab*		NS	6.65	0.02		NS
*Totals and ratios*														
TPL	56.23 ± 4.53		58.01 ± 6.89		61.04 ± 8.69		57.81 ± 7.08			NS		NS		NS
TNL	43.77 ± 4.53		41.99 ± 6.89		38.96 ± 8.69		42.19 ± 7.08			NS		NS		NS
PLs	40.02 ± 2.45		44.76 ± 6.86		44.49 ± 6.64		44.91 ± 5.52			NS		NS		NS
CHO/TPL	0.83 ± 0.06		0.67 ± 0.28		0.63 ± 0.28		0.73 ± 0.20			NS		NS	4.19	0.06
SM/CHO	0.17 ± 0.08	**ab**	0.10 ± 0.06	**b**	0.27 ± 0.11	**a**	0.11 ± 0.06	**b**		NS	6.38	0.02		NS
AN/ZWIT	0.35 ± 0.05	**b**	0.48 ± 0.19	**a**	0.38 ± 0.08	**b**	0.32 ± 0.09	**b**	6.37	0.02	7.10	0.02		NS
SE/CHO	0.60 ± 0.10	**b**	1.08 ± 0.55	**a**	0.71 ± 0.30	**b**	0.58 ± 0.07	**ab**	6.13	0.02	28.16	0.00	6.57	0.02

*Note*: Data are presented as mean ± SD for 5–6 animals per group. Different letters in each row indicate statistical differences at *p* < 0.05 (bold) or 0.05 < *p* < 0.1 (italic).

Abbreviations: Lipid names: CB, cerebrosides; CHO cholesterol; LPC, lysoglycerophosphatidylcholine; MAG, monoacylglycerol; PC, glycerophosphatidylcholine; PE, diacylglycerophosphatidylethanolamine; PG, glycerophosphatidylglycerol; PI, glycerophosphatidylinositol; PLs, Total phospholipids; PlsEtn(a), glycerophosphatidylethanolamine plasmalogens type a; PlsEtn(b), glycerophosphatidylethanolamine plasmalogens type b: 1‐(1Z‐alkenyl),2‐acylglycerophosphoethanolamines or PE(P‐); PS, glycerophosphatidylserline; SE, steryl esters; SF, sulphatides; SM, Sphyngomyelin; TNL, Total neutral lipids; Total PE (TPE) = PE+ PlsEtn(a) + PlsEtn(b); TPL, Total polar lipids.

Aging caused a significant change in the lipid profiles of hippocampal lipid rafts under a standard diet. Thus, in 15 months‐old animals a fed control diet (CTRL15M), a significant depletion of DHA, AA, LCPUFA, total n‐9, as well as reduced UI and PI were observed (Table 1). The relevant UI/sat and PI/sat indexes were significantly reduced. These changes were accompanied by reduced cholesterol and sphingomyelin levels, and cholesterol‐to‐phospholipids ratio (CHO/TPL), and increased sterol esters (SE), SE/CHO, and n‐6/n‐3 ratios, as compared to young littermates (Table 2). The fact that the n‐6/n‐3 increases with aging may favor a proinflammatory state in the brain parenchyma (Stables & Gilroy, 2011). Similar results have been shown in hippocampal extracts from aged WT and transgenic APP/PS1 mice (Taoro‐González et al., 2022).

The reduction in CHO/TPL induced by aging was mostly due to increased levels of TPL, and especially of phosphatidylethanolamine (PE) species, including PE itself and some of its plasmalogens‐related species (PlsEtn) (Table 2). A major portion of PE is in the form of PUFA‐enriched plasmalogens (Skowronska‐Krawczyk & Budin, 2020). Plasmalogens are ether‐linked phospholipids and their hydrolysis release dimethylacetals (DMA). Amongst the different DMAs identified in the current study, 16:0 DMA and 18:0 DMA were the most abundant between saturates and 18:1n‐9 DMA and 18:1n‐7 DMA between unsaturated. Although no detailed molecular identification of plasmalogens was obtained in this study, correlation analyses on animals fed standard diet indicated that DMAs were positively associated, potentially bound, to PlsEtn(a) (18:1n‐9 DMA) and PlsEtn(b) (18:0 DMA). Further, it was evident that plasmalogens and non‐plasmalogen phospholipid oppositely associated with saturates 18:0 and 16:0 (i.e. *r* = 0.652; *p* = 0.041 in PE vs *r* = −0.712; *p* = 0.021 in PlsEtn(b)) and with monoenes, mainly 18:1n‐9 (i.e. *r* = −0.628; *p* = 0.051 for PE vs *r* = 0.723; *p* = 0.018 for PlsEtn(b)). Another differential feature of PLs compared to PE was their opposed affinity for main LCPUFA, with PlsEtn(a) and PlsEtn(b) associating positively to DHA and negatively to AA, while PE was unrelated to either LCPUFA. These observations are relevant from the point of view of aging since PE and PlsEtn(a) increase, while PlsEtn(b) reduces, in older animals, therefore favoring n‐3 depletion of lipid rafts under conditions where these fatty acids supply is limited, such is the standard diet. Depletion of plasmalogens has been reported as one of the earliest events in the development of AD (Han, 2005). These results are also relevant from the oxidative stress point of view, as plasmalogens have been proposed to behave as LCPUFA depots, to participate in membrane fusion, and also to act as molecular sinks for ROS species (Brites et al., 2004; Skowronska‐Krawczyk & Budin, 2020).

Hence, our present results confirm previous studies showing that brain aging is accompanied by a reduction of lipid rafts overall unsaturation and peroxydability, as well as related physicochemical properties (i.e. fluidity), in the context of the so‐called “lipid raft aging” (Diaz et al., 2012; Fabelo et al., 2012, 2014). In line with this, age‐related changes in both total lipid abundance and region‐dependent composition have long been observed, with PUFA contents generally dropping during aging (Ledesma et al., 2012; Naudí et al., 2015). However, recent lipidomic studies in whole brain tissue from distinct areas have shown that unsaturation and peroxydability variables either remain stable or may even increase with aging (Mota‐Martorell et al., 2022; Naudí et al., 2015). These paradoxical effects of aging can be explained in terms of region‐specific membrane lipid metabolism, as well as the differential proportions of rafts and non‐rafts domains in bulk brain membranes.

### 2.2 Dietary LCPUFA supplementation modifies the lipid composition of Hippocampal lipid rafts of young and aged mice

The effects of discrete amounts of LCPUFA in the supplemented diet on the lipid composition of hippocampal lipid rafts are shown in Tables 1 and 2. The most obvious effects are the virtual absence of differences in fatty acids from polar lipids and lipid classes between 6 months (LCPUFA6M) and 15‐month‐old animals (LCPUFA15M). Noticeably, raft fatty acid profiles from mice fed the LCPUFA‐containing diet were quite similar to that of 6 months animals fed a standard diet. Indeed, LCPUFA supplementation restored not only DHA contents in lipid rafts, but also levels of saturates (which tended to increase in old standard animals), n‐9 monoenes, total n‐3, and total PUFA, as well as UI, PI, and their ratios to saturates (PISAT and UISAT, respectively). PE, PlsEtn(a), PlsEtn(b), TPE, AN/ZW, and SE/CHO, were also restored in 15‐month‐old mice fed the LCPUFA diet. Overall, these observations reflect the regulatory potential of DHA in setting the physiochemical properties of lipid rafts.

Although older animals fed with LCPUFA‐enriched diets partly recover the fatty profile of control young animals, significant differences remain in specific fatty acids compared to young animals. Thus, n‐6 LCPUFA arachidonic acid (AA, 20:4n‐6) displays the lowest values in LCPUFA groups, in particular in LCPUFA 15M (−18.9%). Likewise, longer chain n‐6 intermediates 22:4n‐6 (adrenic acid) and 22:5n‐6 (DPA), which participate in the biosynthetic pathway of AA, were significantly reduced (22:4n‐6, −26.7%; 22:5n‐6, −99.6%). Further, levels of the n‐6 precursors 18:2n‐6 (linoleic acid) diminished in LCPUFA groups by −30.9%. The two consequences of these changes are the reduction of n‐6 LCPUFA (−17.5%) and the decrease of the n6/n3 ratio (−27.6%) in response to the diet.

Collectively, these data indicate that LCPUFA supply causes the increase of n‐3 LCPUFA and the reduction of n‐6 PUFA in hippocampal raft membranes. These findings are physiologically relevant, since the lower proportion of the n‐6 series, mainly AA, would reduce the potential generation of proinflammatory mediators in the hippocampus (Stables & Gilroy, 2011). Previously, we have observed that dietary n‐3 LCPUFA supply reduced age‐associated signs of neuroinflammation (Taoro‐González et al., 2022).

### 2.3 Multivariate analyses, main effects and diet‐aging interactions

**FIGURE 1:**
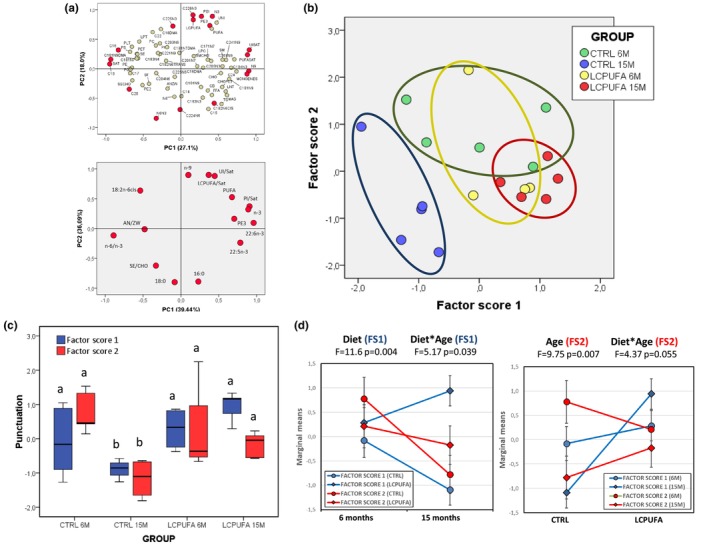
Multivariate assessments of lipid contents of hippocampal lipid rafts. (a) Scatterplot for principal components 1 and 2 showing all lipid variables (upper plot) and selected variables according to their communalities (lower plot). The percent of total variance explained by each component is indicated in parentheses. (b) Factor scores plot for all cases in the study, with representation of group belongings within ellipses. (c) Box‐plots for factor scores 1 and 2. Different letters (a, b) for each factor score indicate statistical differences at *p* < 0.05. (d) Lines plot for marginal means of factor scores 1 and 2 from control (CTRL) and supplemented (LCPUFA) diets in young (6 months) and old (15 months) groups.

Multivariate assessments of lipid fingerprints in both animal and human membrane preparations are accurate (Díaz et al., 2016; Fabelo et al., 2012, 2014). Using an approach based on principal component analyses (PCA), we show here that 13 out of 86 lipid variables in two principal components (Figure 1a) can explain 75.54% of the total variance (PC1 39.44% and PC2 36.09%). Plotting factor scores show that lipid fingerprints of LCPUFA6M and LCPUFA15M groups nearly overlapped, while CTRL6M mice exhibited a high degree of intersection with LCPUFA‐fed animals (Figure 1b). This agrees with the differences in lipid classes and ratios compared to LCPUFA‐fed mice (i.e. SM, PlsEtn(b), PC, PLT, Anionic/Zwitterionic, and SM/CHO) summarized in Table 2. Remarkably, the CTRL15M group segregated and appeared as a differentiated cluster (Figure 1b). One‐way ANOVA indicates that factor loadings for PC1 significantly differ between groups, with LCPUFA‐enriched groups behaving as a homogeneous cluster, similar to the CTRL6M, but not CTRL15M, groups (Figure 1c). In the case of factor score 2, differences were only observed between 6M and 15M control groups.

Two‐way ANOVA of factor scores revealed a main effect of diet on factor score 1 (*F* = 11.60; *p* = 0.004) as well as a significant interaction with age (*F* = 5.17; *p* = 0.039), while for factor score 2, the main effect was age (*F* = 6.01; *p* = 0.007) and potential interaction with diet (*F* = 4.37; *p* = 0.055) (Figure 1d). Exploration of lipid variables showed that those exhibiting the highest communalities in PC1 were mostly modified by diet as the main factor (i.e. n6/n3, 22:6n‐3, 22:5n‐6, 20:4n‐6, PI/Sat, n‐3 series, 22:5n‐3, PlsEtn(b)), and fully or partially restored in older animals by administration of the LCPUFA‐containing diet, depending on their interaction with age (i.e. LCPUFA, n‐3 series, n‐6 series, PI/SAT, SE/CHO). Conversely, some of the variables related to PC2 (SM, TPE, SE, SM/CHO) were mostly modified by age and underwent irreversible age‐associated changes. These observations are biophysically important since the long acyl sphingosine base in SM, and the polar ethanolamine head group of TPEs (including plasmalogens) are largely determinants of interfacial bilayer interaction and lateral packing (Lorent & Levental, 2015) and may predict higher viscosity in age‐associated changes in lipid rafts (see below).

**FIGURE 2:**
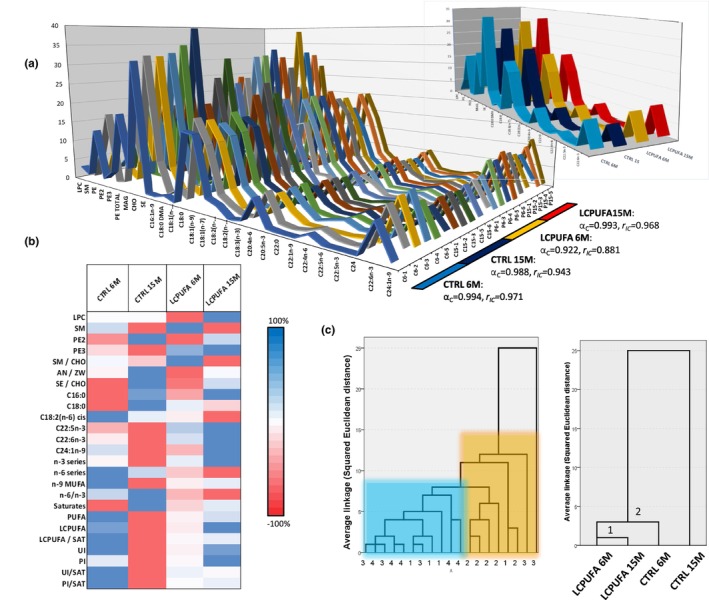
Lipid profiles and cluster analyses of hippocampal lipids in lipid rafts. (a) Reliability analyses of selected variables from PCA. Each line in the plot represents the lipid profile of each individual. Group average lipid profiles are shown in the inset plot. *α*_*C*_: Cronbach's alpha. *r*_IC_: Intraclass correlation. (b) Heatmap for percent changes of selected lipid variables in the four experimental groups. Each cell in the heatmap indicates percent variation (±100%) within each lipid variable. (c) Hierarchical cluster analyses from individuals shown in a (left dendrogram) and from group averages (right dendrogram). 1, 2, 3 and 4 refers to CTRL 6M, CTRL 15M, LCPUFA 6M and LCPUFA 15M, respectively.

We next checked for internal consistencies of the four experimental groups by means of Cronbach's alpha (*α*_*C*_) and Intraclass correlation (*r*_IC_). For this purpose, we used all original lipid variables (fatty acids and lipid classes) within each group. The results show significant *α*_*C*_ and *r*_IC_ values above 0.98 and 0.92, respectively, indicating a very high consistency of cases within each group (Figure 2a). Next, Lipid variables with high loadings in PCA were also analyzed collectively for their intergroup percent differences and heatmap represented (Figure 2b). Overall, each group was characterized by a singular heat pattern in which subtle intergroup differences become maximized. Using these data, we performed hierarchical cluster analyses for Euclidean distances (Figure 2c). The analyses showed that most individuals from groups LCPUFA 6M, and LCPUFA 15M clustered together, in close proximity with most CTRL 6M cases (Figure 2c, left dendrogram), and separated from all CTRL 15M individuals. Using group averages (Figure 2c, right dendrogram), two related clusters were obtained. The closest cluster is formed by LCPUFA groups, which associate in a higher cluster to CTRL 6M group and are totally segregated from CTRL 15M group. These results are in close agreement with the PCA results described above.

Finally, it is noteworthy that hippocampal lipid rafts from 6‐months animals fed standard and LCPUFA‐enriched diets exhibit nearly identical fatty acid profiles, despite large differences in n‐3 PUFA supply (Table S1). However, several metabolic correlates may be inferred from the representation of minor fatty acids in lipid rafts. This is the case of 18:3n‐3, 18:4n‐3, and 18:2n‐6cis (Table 1). These fatty acids are located at the entry point of LCFUFA biosynthesis, and their contents are considerably reduced (or absent in the case of 18:3n‐3 under standard diet) in lipid rafts from aged animals. Their consistent reduction in older animals indicates that even when LCPUFA are provided in the diet, biosynthetic pathways from n‐3 and n‐6 PUFA precursors remain functional as a mechanism to provide additional supply to the brain. Further, the facts that 20:5n‐3 (EPA) and 22:5n‐3 are present in significant amounts in animals receiving the LCPUFA diet (Table 1), independently of age, suggests an efficient mechanism of n‐3 LCPUFA storing in hippocampal membranes. Thus, when diets lack n‐3 LCPUFA, such the standard diet used here, long chain n‐3 and n‐6 fatty acids are synthesized from their n‐3 and n‐6 precursors, 18:n‐3 and 18:2n‐6, respectively, while such metabolic processes are apparently halted or reduced to a minimum in response to the n‐3 LCPUFA containing diet, favoring instead the transport of the longer chain molecule across the blood–brain barrier. This is physiologically relevant, since nervous tissue, in particular hippocampus, does express the genetic and biochemical biosynthetic machinery for PUFA elongation and desaturation (Alessandri et al., 2011; Díaz et al., 2016). A second metabolic consequence of n‐3 LCPUFA supply is the presence of significant amounts of 18:4n‐3 and 22:5n‐3 in LCPUFA groups, but virtually absent in CTRL groups, reflecting a retroconversion mechanism from excess DHA in response to high bioavailability, as reported in other preparations (Brenna, 2019).

### 2.4 Changes in lipid rafts microviscosity and estimated fluidity

**FIGURE 3:**
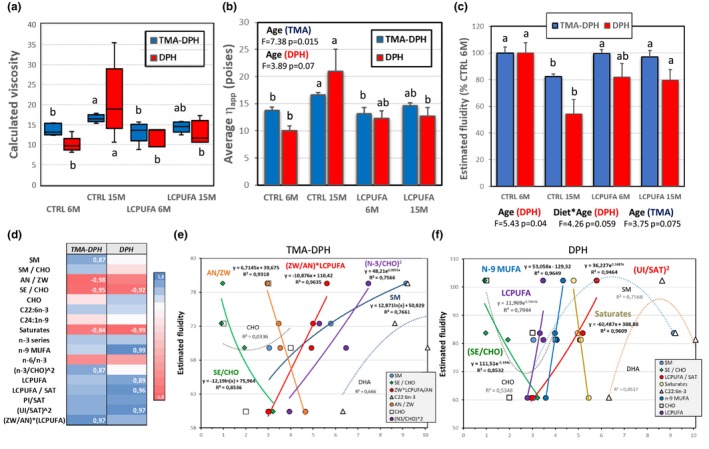
Microviscosity and fluidity analyses of hippocampal lipid rafts in the four experimental groups. (a) Box‐plot for calculated microviscosities for TMA‐DPH and DPH fluorescent probes. (b) Bar plot for mean apparent microviscosities (*η*_app_). (c) Estimated fluidities from the membrane plane (TMA‐DPH) and the hydrophobic region (DPH) relative to 100% in 6 months control group (CTRL 6M). (d) Heatmap for correlation coefficients (***r***) of the linear relationships between lipid variables and estimated fluidities. Highly significant ***r*** values are highlighted in bold numbers. (e, f) Bivariate relationships between different lipid variables and estimated fluidities as determined by TMA‐DPH (e) and DPH (f) probes. Regression equations of best fits and determination coefficients (*R* ^2^) are indicated for each solid line. Dotted lines indicate not significant polynomial relationships between relevant lipid variables (CHO, SM, DHA) and fluidity. Different letters in panels a–c indicate statistical differences at *p* < 0.05.

Fluidity of membranes determines the ease of protein interactions at the initial stages of signaling pathways, but also for conformational changes required for activation of membrane‐buried protein, that is, ligand‐induced receptor activation. LCPUFA are particularly important in setting membrane fluidity. Therefore, we have estimated membrane microviscosity as inferred from two fluorescent probes, namely, TMA‐DPH and DPH, targeting different regions of the membrane. TMA‐DPH is stabilized at the polar head group region of the membrane in the water interface, while DPH localizes within the hydrocarbon chain core of the membrane (do Canto et al., 2016). We detected significant changes in the microviscosity of lipid rafts both at the membrane plane (TMA‐DPH) and the hydrophobic core (DPH) of the lipid bilayer. As seen in Figure 3a, estimated microviscosities were significantly higher in CTRL15M for the two fluorescent probes than in any other groups. No differences were observed between 6M and 15M LCPUFA groups which, in turn, were similar to 6M control mice. Average apparent microviscosities (*η*_app_) determined at the membrane plane and hydrophobic core were very similar in LCPUFA groups (Figure 3b). Conversely, for control animals, *η*_app_ for TMA‐DPH was higher than for DPH at the age of 6 months, but aging caused the opposite effect, with higher *η*_app_ values at the hydrophobic core (Figure 3b). Paralleling these observations, lipid raft fluidity as estimated from TMA‐DPH values, were significantly reduced in CTRL 15M group compared to all other groups, which, in turn, did not differ between each other (Figure 3b). The fact that changes in lipid raft fluidity do not exactly mirror the variations of DHA or LCPUFA contents in lipid rafts indicates that the membrane order state of lipid rafts is regulated by changes in other lipid species and relative proportion. Accordingly, we have found that relationships between lipid rafts fluidity and lipid groups differed depending on the region of the membrane (Figure 3d). Thus, at the membrane‐water interface, estimated fluidity was negatively associated with the ratio Anionic/Zwitterionic phospholipids (*r* = −0.982), SE/CHO (*r* = −0.945), saturates (*r* = −0.838), and positively to SM (*r* = 0.867), (n‐3/CHO)^2^ (*r* = 0.870), being the best correlation to fluidity that of [Zwitterionic/Anionic]*LCPUFA (*r* = 0.965) (Figure 2e). This later relationship [ZW/AN]*LCPUFA is particularly relevant, since phospholipid headgroup composition affects lateral domain packing, being ethanolamine phospholipids the species with largest effect in membrane ordering amongst phospholipids (Lorent & Levental, 2015). Interestingly, membrane fluidity was less correlated to DHA (*r* = 0.718) or n‐3 LCPUFA (*r* = 0.721) and none to cholesterol (*r* = 0.243) on their own, but their significance in setting membrane fluidity increased considerably when related to other lipids (Figure 3e). The general view of these observations is that surface membrane fluidity is mainly related to polar lipids (zwitterionic phospholipids and SM) and the relation of LCPUFA‐to‐cholesterol. Conversely, fluidity at the hydrophobic core was highly correlated to n‐9 MUFA (*r* = 0.982), LCPUFA/SAT (*r* = 0.937), and (UI/SAT)^2^ (*r* = 0.948), and negatively to saturates (*r* = −0.980) (Figure 3f), hence with the physical properties of saturated, mono‐, and polyunsaturated fatty acids. Therefore, we conclude that despite the significant increase in DHA content in lipid raft following n‐3 LCPUFA supplementation, membrane composition is readapted by lipid remodeling to preserve lipid rafts fluidity within physiological values.

These observations indicate a complex scenario well beyond discrete variations of conventional fluidity‐related lipids mostly used in model membranes. The present results show that aging is associated with a decline in the efficiency of homeostatic mechanisms to preserve membrane physicochemical properties. This effect has been demonstrated to represent an early event in the development of Alzheimer's disease (Díaz & Marin, 2021). Indeed, the alteration of biophysical properties of lipid rafts correlates with increased accumulation of amyloidogenic machinery in the human brain cortex (Díaz et al., 2015; Lee et al., 2011). Further, the fact that n‐3 LCPUFA dietary supplementation restores physiological values for lipid raft fluidity demonstrated here, is in close agreement with experimental and epidemiological data showing the beneficial effects of dietary supply of n‐3 polyunsaturated fatty acids a as nutritional strategy to prevent early AD events (Huang, 2010; Solfrizzi et al., 2018).

Finally, lipid packing controls the lateral pressure profiles of membrane domains by the combination of opposed attractive forces (mostly within the hydrophobic core) and repulsive forces (at the head groups) which are balanced in the bilayer structure. Lateral pressure acts on membrane‐buried proteins depending on the lipid composition (Lorent & Levental, 2015). Therefore, this physical parameter not only modulates protein function by affecting the equilibrium in protein conformations but also by favoring or dampening interactions in protein clusters. According to our data, lateral pressure is expected to decrease in hippocampal lipid rafts during control aging as a result of the balance between reduction of UI (attractive forces) and augmented PEs (repulsive forces). At domain scale, lateral forces may also participate in the steady‐state stability of segregated membrane domains, including the different types of lipid rafts and non‐rafts domains.

### 2.5 Changes on lipid rafts molecular hallmarks and Dimensional features

**FIGURE 4:**
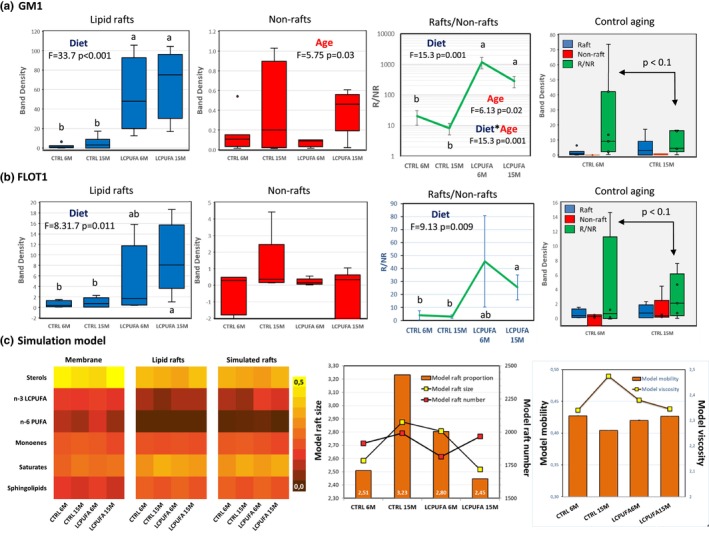
Distribution of lipid rafts markers GM1 (a) and flotilin‐1 (FLOT1, b) in lipid rafts and non‐rafts. Line plots panels in a and b display mean ± SEM raft‐to‐non rafts (R/NR) values in each experimental group. Note the logarithmic scale for GM1 R/NR representation. Rightmost panels in a and b shows a detailed analyses of the effect of aging on GM1 and flotilin‐1 contents in lipid rafts, non‐rafts and R/NR ratio in control groups. Different letters in panels a–c indicate statistical differences at *p* < 0.05. (c) Results from mathematical agent‐based simulations. Left panel illustrates a heatmap of group lipid contents in whole membranes, lipid rafts and simulated lipid rafts for the four experimental groups. Predicted lipid rafts sizes, number of lipid rafts and lipid rafts membrane proportions are represented in the middle panel. Right panel shows model predictions for lipid rafts fluidity and viscosity.

We have analyzed the contents of two main hallmark constituents of lipid rafts, namely ganglioside GM1, and flotillin‐1, in response to the different treatments. GM1 is known to be particularly enriched in the outer leaflet of neuronal lipid rafts. Indeed, the results shown in Figure 4a reveal the nearly exclusive presence of GM1 in lipid rafts compared to non‐rafts, with R/NR (raft‐to‐non‐rafts ratio) values ranging from 10 to 1000. The most evident effect in these experiments was the dramatic increase in R/NR values (500–1000 fold) in n‐3 LCPUFA groups compared to control groups, which was totally attributable to lipid rafts enrichment since non‐rafts GM1 contents did not change between groups. The n‐3 LCPUFA‐driven enrichment of GM1 is likely due to higher mobilization of gangliosides to membrane rafts. To our knowledge, this is the first report showing the enormous impact of DHA on ganglioside contents in lipid rafts. It may be tantalized that GM1 incorporation into rafts derives from non‐rafts membrane reservoirs. This GM1 increase might not be reflected in the corresponding reduction of non‐rafts content due to their much larger proportion as compared to lipid rafts. Alternatively, smaller lipid rafts might cluster together into larger lipid rafts, with proportional increase in total GM1 contents, as a consequence of n‐3 LCPUFA‐induced changes in physicochemical properties of the lipid bilayer. This later hypothesis is supported by evidence from Shaikh et al. (2009) showing that DHA, but not EPA, increased raft size while diminishing raft clustering. Other studies have proposed that enhanced raft formation could be explained by the low affinity of DHA for cholesterol, which effectively causes the clustering and coalescence of cholesterol‐rich domains (Santos et al., 2016; Shaikh et al., 2009).

The enrichment of GM1 in lipid rafts contributes to their stabilization. Thus, SM and gangliosides in nerve cells are rich in saturated fatty acids, whose chains are extended and ordered inside the bilayer and can interact tightly with cholesterol, in brain cell lipid rafts. The close interaction of the planar α‐face of the cholesterol molecule with the ordered acyl chains of Sm and glycosphingolipids within the hydrophobic region promotes additional stabilization of the liquid‐ordered phase (Grassi et al., 2020). A further driving force for domain segregation is represented by the bulkiness of the hydrophilic headgroups of glycosphingolipids and its potential to establish strong conformational associations in glycolipid‐enriched clusters (Grassi et al., 2020). These features make ganglioside GM1 a key factor for the stabilization of DHA‐enriched raft domains resulting from n‐3 LCPUFA supplementation observed here.

Besides their contribution to phase separation and lateral organization of membrane domains, GSLs, and gangliosides in particular, are involved in driving the compartmentalization of “raftophilic” membrane proteins involved in neurotrophic actions, multimolecular complexes such as signalosomes, cellular transduction proteins, ion channels, and transporters, etc. (Allen et al., 2007). These observations have been confirmed in an ever‐increasing number of studies, which collectively have led to consider GM1 gangliosides as key factors in neurotrophic and neuroprotective roles (Chiricozzi et al., 2020; Grassi et al., 2020). In order to delve further into the lipid raft modification in response to n‐3 LCPUFA, we also analyzed the contents of flotillin‐1, a scaffold protein is known to associate with lipid rafts and to participate in the domain stabilization of raft resident and transient signaling proteins (Marin et al., 2009; Marin & Diaz, 2018). This scaffolding structure stabilizes its location within membrane rafts, and it is therefore considered a canonical lipid raft marker. Our results indicate that n‐3 LCPUFA treatment brings about a significant increase in flotillin‐1 contents in hippocampal lipid rafts, without changes in non‐raft membranes (Figure 4b). Consequently, the R/NR is increased by 75‐ and 27‐times in 6M and 15M LCPUFA groups, respectively, compared to age‐matched controls.

Together with the results from GM1, these observations demonstrate that LCPUFA induces the reorganization of membrane domains to increase the total membrane surface corresponding to lipid rafts. As the two markers identify targets located at opposite sides of the membrane, and that the response to LCPUFA enrichment was pretty similar, it is concluded that domain reorganization affects both leaflets of the membrane. Taken together, it may be surmised that LCPUFA enrichment leads to the coalescence of smaller lipid rafts into larger lipid rafts (displaying similar membrane order than smaller rafts, but enriched in GM1 and flotillin‐1), in a process thermodynamically driven by the aversion between DHA (more abundant in non‐raft domains, particularly after n‐3 LCPUFA enrichment) and cholesterol (predominant in lipid rafts), leading to higher membrane entropy. Obviously, experimental demonstration of such segregation is technically unfeasible and out of the scope of this study.

Another physiologically relevant outcome obtained in the present study is the effect of aging on GM1 and flotillin‐1 contents in membrane domains in control animals. The results in Figure 4c indicate that GM1 and flotillin‐1 (FLOT 1) are present almost exclusively in lipid rafts from young animals. This is translated into high R/NR ratios for both markers in 6M CTRL mice. However, these ratios drop by 25% in 15M CTRL animals, indicating that aging provokes the reduction of membrane GM1 and flotilin‐1 levels, therefore affecting not only the stability of lipid rafts but also by undermining ligand recognition by raft receptors and the scaffolding function of rafts to promote the recruitment of signaling molecules and receptors (Colin et al., 2016; Westra et al., 2021; Yaqoob & Shaikh, 2010).

### 2.6 Outcomes from the mathematical model

The results from the mathematical model indicate that the effects of aging and n‐3 LCPUFA supplementation can be largely explained in terms of molecular dynamics. Results in Figure 4c (left panel) compare the actual lipid composition and the prediction given by the model in the four experimental groups. It can be observed that the model yielded a good approximation to the lipid composition of lipid rafts, reproducing the proportions of most lipid groups. The model predicts the increase in lipid rafts size, number, and membrane proportion in the CTRL 15M group compared to CTRL 6M. This group also exhibits the lowest mobility (Figure 4c, right panel). Altogether, this prediction indicates that control ageing causes physicochemical changes in lipid rafts which lead to less fluid cholesterol‐ and saturated‐enriched packed membranes which likely coalesce into larger domains by virtue of repelling forces originated in more fluid surrounding unsaturated‐ LCPUFA‐enriched non‐raft membrane. These results agree with our previous simulations of lipid rafts from frontal cortex in aging mice and aged human brains which, in turn, was found to be exacerbated in transgenic APP/PS1 mice and favorable to promote generation of neurotoxic amyloid peptides (Díaz et al., 2015; Santos et al., 2016; Santos & Díaz, 2021). The model also showed that lipid rafts mean size and average rafts numbers displayed opposite outcomes in LCPUFA 6M (increased size) and LCPUFA 15M (increased number) groups (Figure 4c, middle panel). These resulted in a higher raft proportion in LCPUFA 6M compared to LCPUFA 15M. These observations might explain the larger amounts or GM1 and flot‐1 obtained experimentally, which increased in LCPUFA groups compared to control lipid rafts. Both, augmented size or increased number, in terms of raft proportion are expected to enrich levels of raft markers lipid rafts fractions. Paralleling these observations, no changes were detected for mobility, which may be assimilated to raft fluidity, or estimated viscosity (Figure 4c, right panel) between LCPUFA groups. This is in agreement with the expected behavior of raft proportion (area*number) of lipid rafts with a given membrane order.

Finally, the results of simulated raft mobility/viscosity (Figure 4c, right panel) were consistent with the determinations in the hydrophobic core of the membrane (DPH probe in Figure 3c) rather than in the membrane plane (TMA‐DPH in Figure 3c). At the membrane core, lipid packing is mostly determined by the formation of Van der Waals forces between acyl chains, which expectedly will be larger the lower the UI/SAT ratio, as shown in Figure 3d. Conversely, the model fails at estimating membrane surface fluidity, a drawback that can be interpreted as it ignores essential interactions such as the effects of phospholipids head groups (i.e. ethanolamine) or hydrophobic mismatching between bilayer leaflets.

### 2.7 Changes in hippocampal glutamate receptors in membrane domains

**FIGURE 5:**
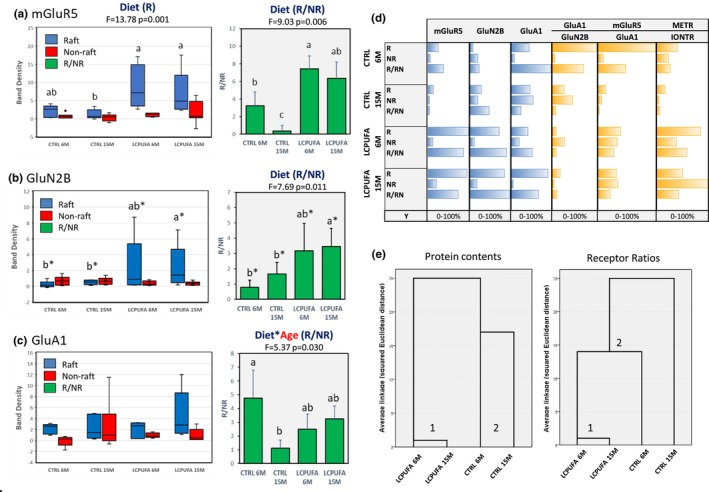
Membrane domain distributions of metabotropic and ionotropic glutamate receptor in hippocampal membrane extracts. (a) Box‐plots for mGluR5 protein presence in lipid rafts and non‐rafts in the four experimental groups. Mean ± SEM for R/NR ratio is illustrated in bar charts (right panel). (b, c) Box‐plots for ionotropic NMDA‐R GluN2B subunit protein (b) and ionotropic AMPA‐R GluA1 subunit (c) in lipid rafts and non‐rafts in the four experimental groups. Mean ± SEM for R/NR ratios are illustrated in bar charts (right panels). In each panel, different letters indicate statistical differences with *p* < 0.05 or 0.05 < *p* < 0.1 (indicated with letter asterisks). (d) Comparative bar plots for fractional (0%–100%) composition of the three types of glutamate receptors, and the ratios AMPA‐R/NAMDA‐R, mGluR5/AMPA‐R, and metabotropic/ionotropic receptors (METR/IONTR) in the two membrane domains and their relative proportions. (e) Hierarchical cluster analyses of subunit protein contents shown in d (left) and receptor ratios (right). Cluster numbers are indicated in the dendrograms.

We investigated the potential changes in the contents of different metabotropic and ionotropic glutamate receptors in hippocampal lipid rafts and non‐raft domains, as well as their relative proportions, in response to aging and LCPUFA supplementation. The results shown in Figure 5 indicate that mGluR5, NMDA‐R GluN2B subunit, and GluA1 AMPA‐R subunit, are expressed both in lipid rafts and non‐rafts domains. Clear effects on protein distribution in response to the LCPUFA diet were observed for mGluR5 and GluN2B in lipid rafts, with no apparent changes in non‐rafts domains (left panels in Figure 5a–c). These results agree with our previous data reported for hippocampal membranes under a similar experimental paradigm (Taoro‐González et al., 2022). However, the analyses of relative raft‐to‐non‐raft (R/NR) proportions revealed that lipid supplementation drives the specific enrichment of mGluR5 and GluN2B (2.1–4.5 fold compared to young animals), but not GluA1, into lipid rafts (right panels in Figure 5a–c). Indeed, two‐way ANOVA revealed no significant interactions between diet and age on R/NR ratios for mGluR5, indicating that LCPUFA supplementation per se is responsible for raft enrichment irrespective of animal aging. Remarkably, a significant reduction in the N/NR ratios for mGluR5 and GluA1 (86.7% and 77.1% compared to 6M mice, respectively) as a consequence of aging is observed in control animals, an effect that likely relates to memory complaints observed during normal aging (Bannerman et al., 2014; Bartsch & Wulff, 2015). Our findings may also relate to potential LTP. As postsynaptic lipid rafts are considered the active site for glutamatergic neurotransmission (Suzuki et al., 2011), the relative abundance of NMDA‐R and AMPA‐R in each domain might reflect the ease to support LTP as a function of age and dietary treatment. As seen in Figure 5d, the GluA1/GluN2B ratio was maximal in CTRL 6M lipid rafts and decreased by 73.1% in CTRL 15M (8.61 vs 2.26). These changes were paralleled by complementary changes in non‐raft fraction, suggesting a redistribution of NMDA and AMPA receptors toward non‐rafts because of aging. The introduction of n‐3 LCPUFA led to reduction of GluA1/GluN2B ratio compared to controls, independently of age, although the ratio values indicated the prevalence of AMPA over NMDA receptors in lipid rafts from LCPUFA groups. Contrary to control animals, non‐rafts in the LCPUFA groups displayed similar values to lipid rafts, suggesting the existence of an inter‐domain equilibrium provoked by changes in membrane physicochemical properties. Therefore, it may be envisaged that LCPUFA drive the synaptic balance between conventional glutamatergic neurotransmission and long‐term potentiation in the context of synaptic plasticity.

By analyzing the mGluR5/GluA1 and metabotropic/ionotropic receptors ratios, it is possible to delve into long‐term depression (LTD) regulation (Collingridge et al., 2010; Jin et al., 2007). Our results indicate a dominance of these ratios in lipid rafts compared to non‐rafts in control animals, yet showing a clear reduction by aging, as well as a considerable increase in both fractions by LCPUFA treatment compared to controls. Of note, as in the case of LTP, both LCPUFA groups exhibit a homogeneous distribution of both mGluR5/GluA1 and METR/IONTR ratios, indicating a redistribution of mGluR and AMPA‐R associated to lipid modification of membrane properties.

A more global effect of LCPUFA supplementation on glutamate receptors redistribution was perceived using cluster analyses (Figure 5f). Thus, it can be observed that both protein contents and receptor ratios share a common pattern of association in LCPUFA groups. However, for control groups, protein contents gather together in a second cluster, yet distant, and unrelated to LCPUFA groups (Figure 5f, left dendrogram), while for receptor ratios, CTRL 6M relates to LCPUFA cluster, though at significant distances (Figure 5f, right dendrogram). These results indicate that n‐3 LCPUFA treatment does not exactly revert the redistribution of glutamate receptors caused by aging, despite increasing their contents in lipid rafts.

The significance of these modifications are currently unknown, but clearly suggest an important role of hippocampal membrane lipids, specifically DHA‐containing phospholipids, in the mechanisms underlying glutamatergic neurotransmission, synaptic organization and plasticity, and memory formation (Vallés & Barrantes, 2022; Westra et al., 2021). In this sense, we have observed in these same groups of animals a decline in the outcomes in the recognition memory (novel object recognition, NOR) and in spatial learning and memory (Barnes maze test) in 15 months old control mice compared to CTRL 6M, which were restored upon dietary supplementation with LCPUFA (Taoro‐González et al., 2022).

In agreement with our findings, it has been shown that dissociated neuronal cultures exposed to exogenous DHA exhibit increased levels of both AMPA‐R and NMDA‐R subunits along with enhanced spontaneous glutamatergic synaptic activity (Cao et al., 2009). Also, in the same study, dietary n‐3 fatty acid deprivation during development resulted in marked decreases of hippocampal levels of glutamate receptor subunits and impaired LTP (Cao et al., 2009). More relevant from the synaptic plasticity point of view, are the studies using dietary supplementation or deprivation of DHA intake. For instance, in old rats, administration of a DHA‐supplemented diet restores the age‐related impairment of LTP (McGahon et al., 1999) and, in young mice, maternal dietary deprivation of DHA leads to inhibited induction of LTP (Cao et al., 2009). Moreover, in our previous study using the same experimental paradigm, we demonstrated that LCPUFA enrichment improved the memory and learning deficits in older control 15M mice, but outcomes from control 6M mice were indistinguishable from 6M or 15M LCPUFA groups (Taoro‐González et al., 2022), suggesting that LCPUFA treatment efficiently prevents age‐associated hippocampal‐dependent memory decline, but do not promote any further enhancement of cognitive abilities.

### 2.8 Changes in hippocampal Acid‐sensing ion channels (ASIC) in membrane domains

Further to glutamate receptors, other membrane channels have been shown to participate in synaptic maintenance. Acid‐sensing ion channels (ASICs) are known to have important roles in modulating the functioning and maintenance of glutamatergic synapses implicated in synaptic plasticity in mouse models of behavior and also in neurodegenerative diseases (Wemmie et al., 2013). In the CNS, the ASIC1a subunit is largely required for acid‐evoked currents as heteromeric complexes formed by association between ASIC1 and ASIC2 subunits, whose expression is promoted by ASIC2 (Wu et al., 2016).

**FIGURE 6:**
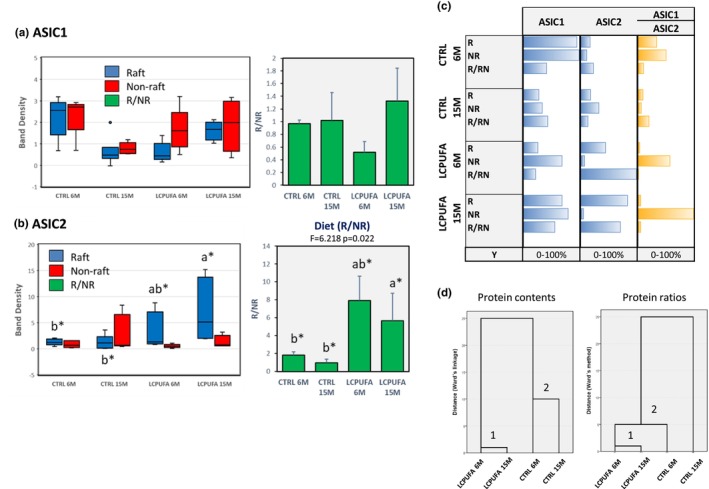
Membrane domain distributions of acid‐sensing ion channels (ASIC) 1 and 2 in hippocampal membrane extracts. (a) Box plots for ASIC1 protein presence in lipid rafts and non‐rafts in the four experimental groups. Mean ± SEM for R/NR ratio is illustrated in bar charts (right panel). (b) Box‐plots for ASIC2 in lipid rafts and non‐rafts in the four experimental groups. Mean ± SEM for R/NR ratios are illustrated in bar charts (right panels). In each panel, different letters indicate statistical differences with 0.05 < *p* < 0.1 (indicated with letter asterisks). (c) Comparative bar plots for fractional (0%–100%) composition of the three types of ion channels, and the ratios ASIC1/ASIC2 in the two membrane domains and their relative proportions. (d) Hierarchical cluster analyses of protein contents shown in c (left) and their ratio (right). Cluster numbers are indicated in the dendrograms.

Therefore, we also assessed the membrane domain contents of acid‐sensing ion channels, ASIC1 and ASCI2, in hippocampal preparations. Results are shown in Figure 6. Show that both channels were similarly expressed in lipid rafts and non‐rafts from control groups, with ASIC1 being twice more abundant than ASIC2 in 6M control animals (Figure 6a,b). Aging caused a reduction in ASIC1 in both raft and non‐raft fractions in the control group, though without affecting the R/NR ratio (Figure 6a, right panel). No effect of aging was observed for ASIC1 or ASIC2 in LCPUFA groups. However, unlike ASCI1, ASCI2 exhibited a diet‐dependent increase in lipid raft contents (3.1–4.3 fold compared to 6M control animals), but not in non‐rafts, resulting in significantly higher R/NR ratios in the hippocampus of LCPUFA supplemented animals (Figure 6b, right panel). As overall changes in ASIC2 were not followed by their reduction in non‐raft, its increase in lipid rafts should be secondary to augmented trafficking from intracellular vesicles enriched in ASIC2‐containing raft‐like membranes, in response to increased n‐3 LCPUFA availability. In line with this, recent studies have demonstrated the selective surface targeting of ASIC2 subunits from ER to modify the formation of heteromeric complexes with ASIC1a (Kweon et al., 2016). Interestingly, comparative analyses of subunits ratio have revealed that the hippocampus exhibits the lowest ASIC1/ASIC2 ratio amongst different brain areas, including cerebral cortex (Wu et al., 2016).

As mentioned before, acid‐evoked currents require the association between ASIC1 and ASIC2 subunits. The proportions of ASIC1 to ASIC2 shown (Figure 6c) indicate that LCPUFA enrichment considerably modifies the ASIC1/ASIC2 ratio with reduction to a minimum in lipid rafts and increase to maximum in non‐rafts. In order to obtain a general view of changes in hippocampal acid‐sensing ion channels induced by aging and dietary LCPUFA, we performed hierarchical cluster analysis. As shown in Figure 6d, two clusters can be identified for ASIC1 and ASIC2 in both membrane domains, corresponding to control and LCPUFA groups (Figure 6d, left dendrogram). However, the analyses of domain proportions ASIC1‐to‐ASIC2 (an approximation to heteromer structure) reveals that LCPUFA groups cluster together in close proximity with CTRL 6M (Figure 6d, right dendrogram). CTRL 15M group exhibits the largest disparity, locating outside the cluster. Of note, the pattern of ASIC association is quite similar to that observed for glutamatergic receptors, indicating that lipid changes participate in lipid raft protein redistribution to adapt physiological ratios required for synapsis.

## 3 CONCLUSIONS

Dietary enrichment of lipid rafts with n‐3 LCPUFA partly restores the molecular and biophysical alterations associated with normal aging, especially with regards to fatty acids and related parameters, as well as membrane fluidity. However, LCPUFA enrichment also modifies the molecular structure and dimension of lipid rafts, the biophysical properties, and the domain redistribution of glutamate receptors and acid‐sensing ion channels, which likely impact in the functionality of lipid rafts in hippocampal synaptic plasticity and memory formation.

## 4 MATERIALS AND METHODS

### 4.1 Animals and treatments

As the mean lifespan of C57BL/6 mice varies between 27 and 30 months, we selected two age groups: 6 months old (20%–22% strain longevity) and 15 months old (50%–55% strain longevity). The experimental design included four groups: a 6‐month‐old fed with a control diet (CTRL 6M), 6‐month‐old fed with a n‐3 LCPUFA‐enriched diet (LCPUFA6M), 15‐month‐old fed with a control diet (CTRL 15M) and 15‐month‐old fed with a n‐3 LCPUFA‐enriched diet (LCPUFA15M). All individuals (≈160 mice) were unmated females. The cohorts were housed together and maintained under standard animal house conditions, a 12 h light–dark cycle, and had ad libitum food and water. All procedures were performed in accordance with local and national guidelines (European Council Directive 86/602/EEC) and was approved by the institutional Research Ethics and Animal Welfare Committee (CEIBA 2019‐0346). Control diet included a standard Teklad Global 14% Protein Rodent Maintenance Diet by Envigo. n‐3 LCPUFA enriched diet was supplemented with Eupoly‐3 DHA oil by Biosearch Life, containing a 2.1:1 EPA‐to‐DHA ratio enrichment, added to the control diet during up to 6 (LCPUFA 6M) and 12 (LCPUFA 15M) weeks before culling. Detailed composition of diets is shown in Table S1.

### 4.2 Total hippocampal lysate and lipid raft extraction

Animals were sacrificed by cervical dislocation. Hippocampi were dissected, frozen in liquid nitrogen, and maintained at −80°C. Hippocampal tissue was homogenized in 1 mL of ice‐cold buffer A (150 mM NaCl, 10 mM MgCl2, 50 mM Tris/HCl pH 8.0) with 1% Triton X‐100, 5% glycerol, and 5 mM β‐mercaptoethanol, proteases and phosphatase inhibitors (1 mM PMSF and complete cocktail, 20 mM NaF, 1 mM Na3VO4). Procedures were performed as previously described (Fabelo et al., 2012, 2014). Briefly, hippocampus homogenates were processed in sucrose gradient and ultracentrifuged (150,000 ***g*** for 18 h at 4°C in a Beckman SW41Ti rotor). Twelve 1 mL fractions were extracted and Lipid rafts were collected from fractions 2 and 3. The 13th fraction (non‐raft fraction) was reconstituted by sonication in buffer A.

### 4.3 Lipid analyses

Lipid composition of hippocampal homogenates and lipid raft fractions were analyzed according to reported methods for these preparations (Fabelo et al., 2014; Martín et al., 2006). Briefly, total lipids were extracted with chloroform/methanol (2:1 v/v) containing butylated hydroxytoluene (0.01%). Then, the organic solvent was evaporated and the lipid content was determined and stored at −20°C. Fatty acids were extracted after acid‐catalyzed transmethylation and the resultant FAMEs (fatty acid methyl esters) and DMAs (dimethylacetals) were purified by thin layer chromatography (TLC), and quantified by gas chromatography using a TRACE GC Ultra equipped with a flame ionization detector (Thermo Fisher Scientific, Massachusetts, USA). Individual FAME and DMA were identified by reference to a multi‐standard mixture (Supelco PARK, Bellefonte, USA), and confirmed using a DSQ II mass spectrometer (Thermo Fisher Scientific, Waltham, MA, USA). Lipid classes were separated by one‐dimensional double development high‐performance thin layer chromatography (HPTLC). Solvent systems were: (1) methyl acetate/isopropanol/chloroform/methanol/0.25% KCl (5:5:5:2:1.8 volume basis) for separation of polar lipid classes, and (2) hexane/diethyl ether/acetic acid (22.5:2.5:0.25 volume basis) for separation of neutral lipid classes. Individual lipid classes were quantified by scanning densitometry using a Shimadzu CS‐9001PC dual wavelength spot scanner.

### 4.4 Microviscosity analysis

Lipid raft microviscosities were determined from steady‐state fluorescence anisotropies of lipid‐soluble probes DPH (diphenylhexatriene) and its cationic derivative TMA‐DPH (trimethylammonium diphenylhexatriene), in purified lipid rafts preparations, following the procedures described in detail in our previous studies for brain cortex (Diaz et al., 2012; Fabelo et al., 2012). Apparent microviscosities (*η*_app_) were estimated at the membrane plane (from TMA‐DPH probe) and the hydrophobic core (from DPH probe) using Perrin equation on steady‐state anisotropies (Díaz et al., 2015). Estimated fluidities were calculated as the inverse of average *η*_app_ and expressed as percent of 6 months control group. Lineal regression analyses were used to evaluate the relationships between fluidity and lipid variables.

### 4.5 Slot blotting

Two hundred nanograms of total protein from raft and non‐raft fractions were kept on ice were spotted onto a nitrocellulose membrane sealed on a Slot‐blot set‐up (Bio‐Rad). Membranes were blocked with every blot‐blocking buffer (Bio‐Rad, California, US) for 5 min at RT and incubated as follows. The immunodetection of GM1, flotillin‐1, mGluR5, GluN2B, GluA1, ASIC1, and ASIC2 was performed by incubation with specific antibodies (Cholera toxin 1:10,000, Anti‐Flotillin‐1, 1:1000, ab41927; Anti‐Metabotropic Glutamate Receptor‐5, 1:1000, ab76316; Anti‐GluN2B, 1:1000, ab65783; anti‐GluA1, 1:1000, ab109450; Anti‐ASIC1, 1:1000, ab240896, and Recombinant Anti‐ASIC2, 1:1000, ab169768) overnight at 4°C. Afterward, membranes were washed three times with TBS‐T solution, followed by incubation with the corresponding secondary‐HRP antibody. After washing again three times with TBS‐T, membranes were then moved into fresh TBS‐T and the signal was detected with Chemie‐Doc MP Imaging System (Bio‐Rad, California, US). Optical densities were quantified and analyzed using Image Lab software. Band intensities were normalized to α‐tubulin bands, which were used as protein loading control.

### 4.6 Mathematical model

We used an agent‐based mathematical model to predict the 2D mobility of the lipid elements in the cell membrane, the lipid raft composition, and their physical properties. The model was initially designed to explain changes in frontal cortex lipid rafts in WT and APP/PS1 transgenic mice (Santos et al., 2016) and subsequently optimized to predict lipid rafts changes in AD human brains (Santos & Díaz, 2021). Six lipid groups are represented in the model: sterols, DHA, n‐6 LCPUFA, MUFA, SFA, and sphingolipids.

### 4.7 Statistics

Raw data were transformed depending on variable characteristics, and submitted to One‐way ANOVA followed by post‐hoc Tukey's or Games‐Howell tests for multiple comparisons of group differences depending on homocedasticity (Levene's test) or non‐parametric Kruskal–Wallis followed by Mann–Whitney tests. Internal consistency of lipid data series was assessed by Cronback's alpha and intraclass correlation. Influences of main factors (age and diet) and their potential interactions were performed by Two‐way ANOVA. Bivariate relationships were assessed by linear regression and correlation analyses. Multivariate analyses were performed by Principal Component Analyses (PCA) and the factor scores were used to establish group signatures. Hierarchical cluster analysis was used to determine Euclidean distances between groups. SPSS 22.0 (IBM, New York, US) software package was used throughout.

## AUTHOR CONTRIBUTIONS

R.M. designed the study and obtained the financial support; D.P. and C.V.‐B. performed the majority of experiments and gathered data; M.D. did the membrane viscosity analyses; G.S. developed the mathematical model. M.D. and D.P. performed the statistical analyses. M.D. prepared figures and wrote the manuscript together with R.M.; All authors revised the draft and approved the final manuscript.

## FUNDING INFORMATION

This study was supported by the grants ProID2020 807 010075 (ACIISI, Gobierno de Canarias, Spain) and SAF2017‐84454‐R (Ministerio de Ciencia e Innovación, Gobierno de España, Spain).

## CONFLICT OF INTEREST STATEMENT

The authors declare no competing financial interests.

## Supporting information


Appendix S1

## Data Availability

All data generated or analyzed during this study are included in this published article and its Appendix S1. Any reasonable request for additional data will be honored.

## References

[acel13867-bib-0001] Alessandri, J. M. , Extier, A. , Al‐Gubory, K. H. , Langelier, B. , Baudry, C. , LePoupon, C. , Lavialle, M. , & Guesnet, P. (2011). Ovariectomy and 17β‐estradiol alter transcription of lipid metabolism genes and proportions of neo‐formed n‐3 and n‐6 long‐chain polyunsaturated fatty acids differently in brain and liver. The Journal of Nutritional Biochemistry, 22, 820–827. 21129945 10.1016/j.jnutbio.2010.07.005

[acel13867-bib-0002] Allen, J. A. , Halverson‐Tamboli, R. A. , & Rasenick, M. M. (2007). Lipid raft microdomains and neurotransmitter signalling. Nature Reviews. Neuroscience, 8, 128–140. 17195035 10.1038/nrn2059

[acel13867-bib-0003] Bannerman, D. M. , Sprengel, R. , Sanderson, D. J. , Mchugh, S. B. , Rawlins, J. N. P. , Monyer, H. , & Seeburg, P. H. (2014). Hippocampal synaptic plasticity, spatial memory and anxiety. Nature Reviews. Neuroscience, 15, 181–192. 24552786 10.1038/nrn3677

[acel13867-bib-0004] Bartsch, T. , & Wulff, P. (2015). The hippocampus in aging and disease: From plasticity to vulnerability. Neuroscience, 309, 1–16. 26241337 10.1016/j.neuroscience.2015.07.084

[acel13867-bib-0005] Brenna, J. T. (2019). DHA retroconversion revisited: dietary DHA spares endogenous EPA. The American Journal of Clinical Nutrition, 110, 789–790. 31250891 10.1093/ajcn/nqz125

[acel13867-bib-0006] Brites, P. , Waterham, H. R. , & Wanders, R. J. A. (2004). Functions and biosynthesis of plasmalogens in health and disease. Biochimica et Biophysica Acta: Molecular and Cell Biology of Lipids, 1636, 219–231. 10.1016/j.bbalip.2003.12.010 15164770

[acel13867-bib-0007] Canerina‐Amaro, A. , Pereda, D. , Diaz, M. , Rodriguez‐Barreto, D. , Casañas‐Sánchez, V. , Heffer, M. , Garcia‐Esparcia, P. , Ferrer, I. , Puertas‐Avendaño, R. , & Marin, R. (2019). Differential aggregation and phosphorylation of alpha Synuclein in membrane compartments associated with Parkinson disease. Frontiers in Neuroscience, 13, 1–21. 31068782 10.3389/fnins.2019.00382 PMC6491821

[acel13867-bib-0008] Cao, D. , Kevala, K. , Kim, J. , Moon, H.‐S. , Beom Jun, S. , Lovinger, D. , & Kim, H.‐Y. (2009). Docosahexaenoic acid promotes hippocampal neuronal development and synaptic function. Journal of Neurochemistry, 111, 510–521. 19682204 10.1111/j.1471-4159.2009.06335.x PMC2773444

[acel13867-bib-0009] Catalá, A. , & Díaz, M. (2016). Editorial: Impact of lipid peroxidation on the physiology and pathophysiology of cell membranes. Frontiers in Physiology, 7, 1–3. 27713704 10.3389/fphys.2016.00423 PMC5031777

[acel13867-bib-0010] Chiricozzi, E. , Lunghi, G. , Di, B. E. , Fazzari, M. , Sonnino, S. , & Mauri, L. (2020). GM1 ganglioside is a key factor in maintaining the mammalian neuronal functions avoiding neurodegeneration. International Journal of Molecular Sciences, 21, 1–29. 10.3390/ijms21030868 PMC7037093 32013258

[acel13867-bib-0011] Colin, J. , Gregory‐Pauron, L. , Lanhers, M. C. , Claudepierre, T. , Corbier, C. , Yen, F. T. , Malaplate‐Armand, C. , & Oster, T. (2016). Membrane raft domains and remodeling in aging brain. Biochimie, 130, 178–187. 27594339 10.1016/j.biochi.2016.08.014

[acel13867-bib-0012] Collingridge, G. L. , Peineau, S. , Howland, J. G. , & Wang, Y. T. (2010). Long‐term depression in the CNS. Nature Reviews. Neuroscience, 11, 459–473. 20559335 10.1038/nrn2867

[acel13867-bib-0013] Delint‐Ramirez, I. , Fernández, E. , Bayés, A. , Kicsi, E. , Komiyama, N. H. , & Grant, S. G. N. (2010). In vivo composition of NMDA receptor signaling complexes differs between membrane subdomains and is modulated by PSD‐95 and PSD‐93. The Journal of Neuroscience, 30, 8162–8170. 20554866 10.1523/JNEUROSCI.1792-10.2010 PMC2912510

[acel13867-bib-0014] Díaz, M. , Fabelo, N. , Casañas‐Sánchez, V. , Marin, R. , Gómez, T. , Quinto‐Alemany, D. , & Pérez, J. A. (2016). Hippocampal lipid homeostasis in APP/PS1 mice is modulated by a complex interplay between dietary DHA and estrogens: Relevance for Alzheimer's disease. Journal of Alzheimer's Disease, 49, 459–481. 10.3233/JAD-150470 26519437

[acel13867-bib-0015] Díaz, M. , Fabelo, N. , Ferrer, I. , & Marín, R. (2018). “Lipid raft aging” in the human frontal cortex during nonpathological aging: gender influences and potential implications in Alzheimer's disease. Neurobiology of Aging, 67, 42–52. 29627763 10.1016/j.neurobiolaging.2018.02.022

[acel13867-bib-0016] Díaz, M. , Fabelo, N. , Martín, V. , Ferrer, I. , Gómez, T. , & Marín, R. (2015). Biophysical alterations in lipid rafts from human cerebral cortex associate with increased BACE1/AβPP interaction in early stages of Alzheimer's disease. Journal of Alzheimer's Disease, 43, 1185–1198. 10.3233/JAD-141146 25147112

[acel13867-bib-0017] Díaz, M. , & Marin, R. (2021). Lipid rafts and development of Alzheimer's disease. In S. J. Baloyannis (Ed.), Cerebral and Cerebellar Cortex – Interaction and Dynamics in Health and Disease, Ch. 7. IntechOpen. 10.5772/intechopen.94608

[acel13867-bib-0018] Diaz, M. L. , Fabelo, N. , & Marín, R. (2012). Genotype‐induced changes in biophysical properties of frontal cortex lipid raft from APP/PS1 transgenic mice. Frontiers in Physiology, 3, 1–10. 23205014 10.3389/fphys.2012.00454 PMC3506919

[acel13867-bib-0019] do Canto, A. M. T. M. , Robalo, J. R. , Santos, P. D. , Carvalho, A. J. P. , Ramalho, J. P. P. , & Loura, L. M. S. (2016). Diphenylhexatriene membrane probes DPH and TMA‐DPH: A comparative molecular dynamics simulation study. Biochimica et Biophysica Acta, 1858, 2647–2661. 27475296 10.1016/j.bbamem.2016.07.013

[acel13867-bib-0020] Egawa, J. , Pearn, M. L. , Lemkuil, B. P. , Patel, P. M. , & Head, B. P. (2016). Membrane lipid rafts and neurobiology: age‐related changes in membrane lipids and loss of neuronal function. The Journal of Physiology, 594, 4565–4579. 26332795 10.1113/JP270590 PMC4983616

[acel13867-bib-0021] Fabelo, N. , Martín, V. , Marín, R. , Moreno, D. , Ferrer, I. , & Díaz, M. (2014). Altered lipid composition in cortical lipid rafts occurs at early stages of sporadic Alzheimer's disease and facilitates APP/BACE1 interactions. Neurobiology of Aging, 35, 1801–1812. 24613671 10.1016/j.neurobiolaging.2014.02.005

[acel13867-bib-0022] Fabelo, N. , Martín, V. , Marín, R. , Santpere, G. , Aso, E. , Ferrer, I. , & Díaz, M. (2012). Evidence for premature lipid raft aging in APP/PS1 double‐transgenic mice, a model of familial Alzheimer disease. Journal of Neuropathology and Experimental Neurology, 71, 868–881. 10.1097/NEN.0b013e31826be03c 22975585

[acel13867-bib-0023] Favrelière, S. , Stadelmann‐Ingrand, S. , Huguet, F. , De, J. D. , Piriou, A. , Tallineau, C. , & Durand, G. (2000). Age‐related changes in ethanolamine glycerophospholipid fatty acid levels in rat frontal cortex and hippocampus. Neurobiology of Aging, 21, 653–660. 11016534 10.1016/s0197-4580(00)00170-6

[acel13867-bib-0024] Grassi, S. , Giussani, P. , Mauri, L. , Prioni, S. , Sonnino, S. , & Prinetti, A. (2020). Lipid rafts and neurodegeneration: structural and functional roles in physiologic aging and neurodegenerative diseases. Journal of Lipid Research, 61, 636–654. 31871065 10.1194/jlr.TR119000427 PMC7193971

[acel13867-bib-0025] Groc, L. , & Choquet, D. (2020). Linking glutamate receptor movements and synapse function. Science, 368, 1–9. 10.1126/science.aay4631 32527803

[acel13867-bib-0026] Han, X. (2005). Lipid alterations in the earliest clinically recognizable stage of Alzheimer's disease: implication of the role of lipids in the pathogenesis of Alzheimer's disease. Current Alzheimer Research, 2, 65–77. 15977990 10.2174/1567205052772786

[acel13867-bib-0027] Huang, T. L. (2010). Omega‐3 fatty acids, cognitive decline, and Alzheimer's disease: a critical review and evaluation of the literature. Journal of Alzheimer's Disease, 21, 673–690. 10.3233/JAD-2010-090934 20634589

[acel13867-bib-0028] Jin, Y. , Kim, S. J. , Kim, J. , Worley, P. F. , & Linden, D. J. (2007). Long‐term depression of mGluR1 signaling. Neuron, 55, 277–287. 17640528 10.1016/j.neuron.2007.06.035 PMC2063510

[acel13867-bib-0029] Kweon, H. J. , Kim, D. I. , Bae, Y. , Park, J. Y. , & Suh, B. C. (2016). Acid‐Sensing Ion Channel 2a (ASIC2a) promotes surface trafficking of ASIC2b via heteromeric assembly. Scientific Reports, 6, 1–10. 27477936 10.1038/srep30684 PMC4967927

[acel13867-bib-0030] Ledesma, M. D. , Martin, M. G. , & Dotti, C. G. (2012). Lipid changes in the aged brain: effect on synaptic function and neuronal survival. Progress in Lipid Research, 51, 23–35. 22142854 10.1016/j.plipres.2011.11.004

[acel13867-bib-0031] Lee, J. C. M. , Askarova, S. , & Yang, X. (2011). Impacts of membrane biophysics in Alzheimer's disease: From amyloid precursor protein processing to aβ Peptide‐induced membrane changes. International Journal of Alzheimer's Disease, 2011, 134971. 10.4061/2011/134971 PMC3087431 21547213

[acel13867-bib-0032] Lorent, J. H. , & Levental, I. (2015). Structural determinants of protein partitioning into ordered membrane domains and lipid rafts. Chemistry and Physics of Lipids, 192, 23–32. 26241883 10.1016/j.chemphyslip.2015.07.022

[acel13867-bib-0033] Marin, R. , & Diaz, M. (2018). Estrogen Interactions With Lipid Rafts Related to Neuroprotection. Impact of Brain Ageing and Menopause. Frontiers in Neuroscience, 12, 1–18. 29559883 10.3389/fnins.2018.00128 PMC5845729

[acel13867-bib-0034] Marin, R. , Díaz, M. , Alonso, R. , Sanz, A. , Arévalo, M. A. , & Garcia‐Segura, L. M. (2009). Role of estrogen receptor α in membrane‐initiated signaling in neural cells: Interaction with IGF‐1 receptor. The Journal of Steroid Biochemistry and Molecular Biology, 114, 2–7. 19167493 10.1016/j.jsbmb.2008.12.014

[acel13867-bib-0035] Martín, V. , Almansa, E. , Fabelo, N. , & Díaz, M. (2006). Selective polyunsaturated fatty acids enrichment in phospholipids from neuronal‐derived cell lines. Journal of Neuroscience Methods, 153, 230–238. 16337275 10.1016/j.jneumeth.2005.10.019

[acel13867-bib-0036] McGahon, B. M. , Martin, D. S. D. , Horrobin, D. F. , & Lynch, M. A. (1999). Age‐related changes in synaptic function: analysis of the effect of dietary supplementation with omega‐3 fatty acids. Neuroscience, 94, 305–314. 10613520 10.1016/s0306-4522(99)00219-5

[acel13867-bib-0037] Mota‐Martorell, N. , Andrés‐Benito, P. , Martín‐Gari, M. , Galo‐Licona, J. D. , Sol, J. , Fernández‐Bernal, A. , Portero‐Otín, M. , Ferrer, I. , Jove, M. , & Pamplona, R. (2022). Selective brain regional changes in lipid profile with human aging. GeroScience, 44, 763–783. 35149960 10.1007/s11357-022-00527-1 PMC9135931

[acel13867-bib-0038] Naudí, A. , Cabré, R. , Jové, M. , Ayala, V. , Gonzalo, H. , Portero‐Otín, M. , Ferrer, I. , & Pamplona, R. (2015). Lipidomics of human brain aging and Alzheimer's disease pathology. International Review of Neurobiology, 122, 133–189. 26358893 10.1016/bs.irn.2015.05.008

[acel13867-bib-0039] Santos, G. , & Díaz, M. (2021). Dimensional changes in lipid rafts from human brain cortex associated to development of Alzheimer's disease. Predictions from an agent‐based mathematical model. International Journal of Molecular Sciences, 22, 1–14. 10.3390/ijms222212181 PMC8620379 34830060

[acel13867-bib-0040] Santos, G. , Díaz, M. , & Torres, N. V. (2016). Lipid raft size and lipid mobility in non‐raft domains increase during aging and are exacerbated in APP/PS1 mice model of Alzheimer's disease. Predictions from an agent‐based mathematical model. Frontiers in Physiology, 7, 1–13. 27014089 10.3389/fphys.2016.00090 PMC4791387

[acel13867-bib-0041] Shaikh, S. R. , Rockett, B. D. , Salameh, M. , & Carraway, K. (2009). Docosahexaenoic acid modifies the clustering and size of lipid rafts and the lateral organization and surface expression of MHC class I of EL4 cells. The Journal of Nutrition, 139, 1632–1639. 19640970 10.3945/jn.109.108720

[acel13867-bib-0042] Skowronska‐Krawczyk, D. , & Budin, I. (2020). Aging membranes: Unexplored functions for lipids in the lifespan of the central nervous system. Experimental Gerontology, 131, 110817. 31862420 10.1016/j.exger.2019.110817 PMC7877915

[acel13867-bib-0043] Solfrizzi, V. , Agosti, P. , Lozupone, M. , Custodero, C. , Schilardi, A. , Valiani, V. , Santamato, A. , Sardone, R. , Dibello, V. , Di, L. L. , Stallone, R. , Ranieri, M. , Bellomo, A. , Greco, A. , Daniele, A. , Seripa, D. , Sabbà, C. , Logroscino, G. , & Panza, F. (2018). Nutritional interventions and cognitive‐related outcomes in patients with late‐life cognitive disorders: A systematic review. Neuroscience and Biobehavioral Reviews, 95, 480–498. 30395922 10.1016/j.neubiorev.2018.10.022

[acel13867-bib-0044] Stables, M. J. , & Gilroy, D. W. (2011). Old and new generation lipid mediators in acute inflammation and resolution. Progress in Lipid Research, 50, 35–51. 20655950 10.1016/j.plipres.2010.07.005

[acel13867-bib-0045] Suzuki, T. , Zhang, J. , Miyazawa, S. , Liu, Q. , Farzan, M. R. , & Yao, W. D. (2011). Association of membrane rafts and postsynaptic density: Proteomics, biochemical, and ultrastructural analyses. Journal of Neurochemistry, 119, 64–77. 21797867 10.1111/j.1471-4159.2011.07404.x PMC3184177

[acel13867-bib-0046] Taoro‐González, L. , Pereda, D. , Valdés‐Baizabal, C. , González‐Gómez, M. , Pérez, J. A. , Mesa‐Herrera, F. , Canerina‐Amaro, A. , Pérez‐González, H. , Rodríguez, C. , Díaz, M. , & Marin, R. (2022). Effects of dietary n‐3 LCPUFA supplementation on the hippocampus of aging female mice: Impact on memory, lipid raft‐associated glutamatergic receptors and neuroinflammation. International Journal of Molecular Sciences, 23, 1–24. 10.3390/ijms23137430 PMC9267073 35806435

[acel13867-bib-0047] Ulmann, L. , Mimouni, V. , Roux, S. , Porsolt, R. , & Poisson, J. P. (2001). Brain and hippocampus fatty acid composition in phospholipid classes of aged‐relative cognitive deficit rats. Prostaglandins, Leukotrienes, and Essential Fatty Acids, 64, 189–195. 11334555 10.1054/plef.2001.0260

[acel13867-bib-0048] Vallés, A. S. , & Barrantes, F. J. (2022). The synaptic lipidome in health and disease. Biochimica et Biophysica Acta – Biomembranes, 1864, 184033. 35964712 10.1016/j.bbamem.2022.184033

[acel13867-bib-0049] Wemmie, J. A. , Taugher, R. J. , & Kreple, C. J. (2013). Acid‐sensing ion channels in pain and disease. Nature Reviews. Neuroscience, 14, 461–471. 23783197 10.1038/nrn3529 PMC4307015

[acel13867-bib-0050] Westra, M. , Gutierrez, Y. , & MacGillavry, H. D. (2021). Contribution of membrane lipids to postsynaptic protein organization. Frontiers in Synaptic Neuroscience, 13, 1–14. 10.3389/fnsyn.2021.790773 PMC8649999 34887741

[acel13867-bib-0051] Wood, P. L. (2012). Lipidomics of Alzheimer's disease: current status. Alzheimer's Research & Therapy, 4, 5. 10.1186/alzrt103 PMC3471525 22293144

[acel13867-bib-0052] Wu, J. , Xu, Y. , Jiang, Y. Q. , Xu, J. , Hu, Y. , & Zha, X. M. (2016). ASIC subunit ratio and differential surface trafficking in the brain. Molecular Brain, 9, 4. 26746198 10.1186/s13041-016-0185-7 PMC4706662

[acel13867-bib-0053] Yaqoob, P. , & Shaikh, S. R. (2010). The nutritional and clinical significance of lipid rafts. Current Opinion in Clinical Nutrition and Metabolic Care, 13, 156–166. 20010096 10.1097/MCO.0b013e328335725b

[acel13867-bib-0054] Yoon, J. H. , Seo, Y. , Jo, Y. S. , Lee, S. , Cho, E. , Cazenave‐Gassiot, A. , Shin, Y. S. , Moon, M. H. , An, H. J. , Wenk, M. R. , & Suh, P. G. (2022). Brain lipidomics: From functional landscape to clinical significance. Science Advances, 8, eadc9317. 36112688 10.1126/sciadv.adc9317 PMC9481132

[acel13867-bib-0055] Zhang, X. , Han, H. , Ge, X. , Liu, L. , Wang, T. , & Yu, H. (2020). Effect of n‐3 long‐chain polyunsaturated fatty acids on mild cognitive impairment: A meta‐analysis of randomized clinical trials. European Journal of Clinical Nutrition, 74, 548–554. 31804628 10.1038/s41430-019-0544-4

