# Supplementary material for: Molecular and biophysical features of hippocampal “lipid rafts aging” are modified by dietary n‐3 long‐chain polyunsaturated fatty acids
Source: Aging Cell. 2023 May 30;22(8):e13867. doi: 10.1111/acel.13867 (PMC10410061; doi:10.1111/acel.13867)
Supplement: Supplementary file 1 — Appendix S1 [file ACEL-22-e13867-s001.pdf]

Supplementary Table 1. Composition of standard (Control) and n-3 LCPUFA-supplemented diets

**Analytical constituents**	**Control diet (%)**	**n-3 LCPUFA (%)**
Moisture	12.00	12.00
Crude protein	14.50	14.50
Crude fibres	4.50	4.50
Crude ash	4.70	4.70
Crude oil and fats	4.00	4.82
Fatty acids composition		
Total Saturated	0.6	0.6
C16:0 (Palmitic acid)	0.5	0.5
C18:0 (Stearic acid)	0.1	0.1
Total Monounsaturated (MUFA)	0.7	0.7
C18:1n9 (Oleic acid)	0.7	0.7
Total Polyunsaturated (PUFA)	2.1	2.92
C18:2n6 (Linoleic acid)	2.0	2.0
C18:3n3 (Linolenic acid)	0.1	0.1
C20:5n3 (EPA)	--	0.56
C22:6n3 (DHA)	--	0.26
